# Automatic Assessment of Transcatheter Aortic Valve Implantation Results on Four-Dimensional Computed Tomography Images Using Artificial Intelligence

**DOI:** 10.3390/bioengineering10101206

**Published:** 2023-10-16

**Authors:** Laura Busto, César Veiga, José A. González-Nóvoa, Silvia Campanioni, Pablo Juan-Salvadores, Víctor Alfonso Jiménez Díaz, José Antonio Baz, José Luis Alba-Castro, Maximilian Kütting, Andrés Íñiguez

**Affiliations:** 1 Cardiology Research Group, Galicia Sur Health Research Institute (IIS Galicia Sur), 36312 Vigo, Spain; laura.busto@iisgaliciasur.es (L.B.); jose.gonzalez@iisgaliciasur.es (J.A.G.-N.); silvia.campanioni@iisgaliciasur.es (S.C.); pablo.juan@iisgaliciasur.es (P.J.-S.); 2 Cardiology Department, Complexo Hospitalario Universitario de Vigo (SERGAS), Álvaro Cunqueiro Hospital, 36312 Vigo, Spain; victor.alfonso.jimenez.diaz@sergas.es (V.A.J.D.); jose.antonio.baz.alonso2@sergas.es (J.A.B.); andres.iniguez.romo@sergas.es (A.Í.); 3 atlanTTic Research Center for Telecommunication Technologies, University of Vigo, 36310 Vigo, Spain; jalba@gts.uvigo.es; 4 New Valve Technology/Biosensors, 72379 Hechingen, Germany; m.kuetting@nvt-med.com

**Keywords:** transcatheter aortic valve implantation (TAVI), four-dimensional computed tomography (4D-CT), artificial intelligence (AI), fully automatic assessment, device-anatomy characterization

## Abstract

Transcatheter aortic valve implantation (TAVI) is a procedure to treat severe aortic stenosis. There are several clinical concerns related to potential complications after the procedure, which demand the analysis of computerized tomography (CT) scans after TAVI to assess the implant’s result. This work introduces a novel, fully automatic method for the analysis of post-TAVI 4D-CT scans to characterize the prosthesis and its relationship with the patient’s anatomy. The method enables measurement extraction, including prosthesis volume, center of mass, cross-sectional area (CSA) along the prosthesis axis, and CSA difference between the aortic root and prosthesis, all the variables studied throughout the cardiac cycle. The method has been implemented and evaluated with a cohort of 13 patients with five different prosthesis models, successfully extracting all the measurements from each patient in an automatic way. For Allegra patients, the mean of the obtained inner volume values ranged from 10,798.20 mm^3^ to 18,172.35 mm^3^, and CSA in the maximum diameter plane varied from 396.35 mm^2^ to 485.34 mm^2^. The implantation of this new method could provide information of the important clinical value that would contribute to the improvement of TAVI, significantly reducing the time and effort invested by clinicians in the image interpretation process.

## 1. Introduction

Transcatheter aortic valve implantation (TAVI) is a medical procedure for the replacement of the aortic valve using a catheter to deliver and deploy a replacement valve (prosthesis) in the aortic root [1]. In recent years, TAVI has become the preferred treatment for inoperable patients suffering from symptomatic severe aortic stenosis, as well as an alternative to surgical aortic valve replacement in patients with an increased risk associated with surgery [2]. In addition, the procedure is being performed on progressively younger patients [3], significantly expanding the clinical impact of TAVI.

The twenty years of experience of the procedure have provided a consensus regarding the imaging modalities involved in TAVI [4]. Several imaging modalities are used in the different stages of the process (pre-, peri-, and post-TAVI). Pre-procedural planning employs echocardiography and computed tomography (CT) for selecting the access via and dimension of the device suitable for each patient [5]. Intra-procedural implantation uses mainly angiography for real-time navigation, as well as angiography and echocardiography for assessing the implant parameters, once the prosthesis is deployed. Post-procedural clinical follow-up of patients requires the expert use of various imaging modalities, each of which has its own strengths and limitations.

After the procedure, there are still various clinical issues associated with potential complications from TAVI. Mainly, there are four different kinds of complications with important clinical impact: structural valve deterioration, non-structural valve deterioration, thrombosis, and endocarditis [6]. The structural valve deterioration refers to permanent changes in the prosthesis, such as calcification, leaflet fibrosis, or tear, degenerating the prosthesis function, which can lead to an eventual hemodynamic dysfunction. Regarding these kinds of complications, the prosthesis durability has become a major concern, as TAVI is being performed on younger patients [3], and thus it is crucial to focus on the prosthesis durability to reduce possible future complications and reinterventions, which imply higher healthcare costs and higher risks for the patient. Non-structural valve deterioration refers to abnormalities not intrinsic of the prosthesis itself but prosthetic regurgitation, malposition, mismatch with the patient anatomy, or late embolization [6]. Leaflet thrombosis (LT) [7] refers to the formation of blood clots on one or more of the prosthetic valve leaflets, which can lead to valve malfunction or obstruction due to the clots interfering with the movement of the leaflets of the prosthesis. Infective endocarditis (IE) after TAVI is an emerging complication, for which existing data reflect incomplete and diverse incidence. The IE diagnostic in prosthetic valves is based on the Duke modified criteria using 18F-fluorodeoxyglucose positron emission tomography/computed tomography (18F-FDG PET/CT) nuclear imaging [8].

Most of the previously mentioned clinical concerns can be addressed using ECG-gated multidetector CT (MDCT). The employment of time volumetric series generated by new four-dimensional computed tomography (4D-CT) can incorporate the time variable into the analysis. This imaging modality provides the major advantages, as it can provide early detection of post-interventional complications of the prosthesis and the aortic root, and it is superior to echocardiography with respect to the direct detection of LT [9]. Post-TAVI CT can also evaluate the valve alignment, that could be useful information for eventual future coronary interventions [10]. In addition, CT allows accurate geomorphological assessment of the TAVI implant, which can be used to extract features such as prosthesis symmetry, expansion, and depth, which have been demonstrated to be related to the development of LT [11]. Studying the valve alignment, along with further data, can also impact valvular hemodynamics and LT [11,12]. Evaluation of other parameters such as coronary–leaflet distance (for valve-in-valve planning), anterior mitral leaflet displacement, and annular damage is also of major clinical importance.

The measurements that can be extracted from CT imaging enable the characterization of the prosthesis and the implant, meaning the relationship between the device and the patient’s anatomy. Studying the geometrical properties of the prosthesis and the aortic root as 3D structures and analyzing the 2D cross-sections along the prosthesis long (principal) axis may provide useful information about the assessment of the implant. Such assessment can be performed via a visual analysis by a clinician, but this is a tedious and time-consuming task, especially when carried out with different time volumes. In addition, it requires a high level of expertise, making the analysis dependent on the availability of the expert and limiting the capability of analysis, even making it unapproachable in some cases. Therefore, the automation of the interpretation of TAVI images is concerning and could significantly help the clinical practice, minimizing the effort per analysis. Furthermore, the employment of computers and artificial intelligence techniques for image interpretation would provide quantitative information that can be insightful for TAVI assessment [13].

In the literature, there are studies that investigate how the geometric characteristics of prostheses, extractable from 4D-CT scans, may impact potential complications after TAVI [14,15,16]. These geometric features encompass aspects such as the relative positions concerning the prosthesis and the aortic root, asymmetric expansion of the prosthesis, or the deformation of the stent frame. This deformation is typically quantified by extracting the cross-sectional area (CSA) measurements of the stent frame at specific levels, such as the frame inflow, native annulus, or frame outflow. Despite the acknowledgment of the significance of such studies in the literature, it is important to note that they often focus on very specific prosthesis models, primarily the Sapien 3 and Evolut R/PRO and may not provide details regarding their analytical methodologies or acknowledge the use of specific automated tools. Hence, there is interest in designing and developing automated tools capable of conducting this type of analysis using post-TAVI 4D-CT scans.

This work introduces a new method for interpreting 4D-CT studies, providing valuable quantitative information relevant to the assessment of TAVI. This method not only contributes to improving the TAVI procedure but also streamlines the image interpretation process, saving both time and effort. The key highlights of this work include its capability for fully automatic analysis of post-TAVI scans, a practical implementation of the method, and the presentation of successful results obtained from an image dataset collected by the authors. The remainder of the paper is structured as follows: Section 2 describes the dataset and the software tools employed in this work, as well as a detailed explanation of the methodology and some of its applications, Section 3 provides the results obtained with the method implementation for the dataset. The discussion and conclusions of the work are drawn in Section 4 and Section 5, respectively.

## 2. Materials and Methods

### 2.1. Dataset

The images used in this work are 4D-CT studies obtained from 13 patients (84.8 ± 2.44 years, 77.77% female) with severe aortic stenosis and who had previously undergone TAVI. Regarding the prosthesis models, 11 patients received Allegra (Biosensors/NVT GmbH, Hechingen, Germany) prostheses (three of them with a prosthesis diameter of 23 mm, five of 27 mm, and three of 31 mm), and two patients received CoreValve Evolut R (Medtronic CoreValve LLC, Minneapolis, MN, USA) prostheses (with a prosthesis diameter of 26 mm and 34 mm). The images were acquired from consecutive patients at the Department of Cardiology at Álvaro Cunqueiro Hospital, Vigo, Spain, from July 2021 to July 2022. The trial protocol was approved by the Spanish national authorities and ethics committees and by institutional research boards at the participating site. Informed consent was obtained and documented for all patients before conducting any study-related procedures. The 4D-CT studies were acquired using Siemens SOMATOM Drive Equipment. One time sequence has been captured for each patient, each with 10 time volumes over the cardiac cycle. Each time volume contains on average 345.5 slices, with an average slice spacing of 0.56 mm. Each slice is a DICOM image of 256×256 pixels, a pixel spacing of 0.65 mm on average, and a pixel value range of (0, 4095).

### 2.2. Software Tools

The software 3D Slicer (version 5.2.2) [17] is a free, open-source platform for the visualization, processing, and analysis of medical images and computing datasets. One of its main strengths is the high number of extensions available on the platform, as well as the growing community, who make valuable contributions to the development of the platform and of new tools. The software includes a Python environment, where all the platform features are available, which allow researchers to develop and to evaluate new methods and prototypes, as well as distribute them among the community.

### 2.3. Proposed Method

**Figure 1:**
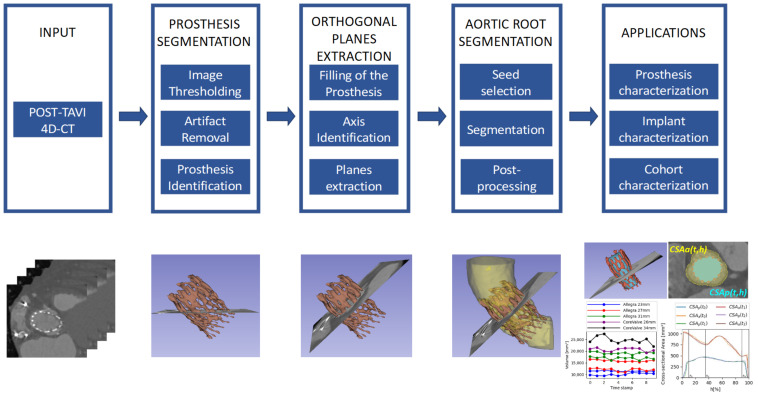
Schema of the pipeline for automatic extraction of TAVI devices and implants proposed in this work.

In this work, we propose a new method, consisting of the blocks included in the diagram in Figure 1, for the extraction of measurements of interest from 4D-CT images to assess the implant after the TAVI procedure in a fully automatic way, allowing a more precise assessment.

**Figure 2:**
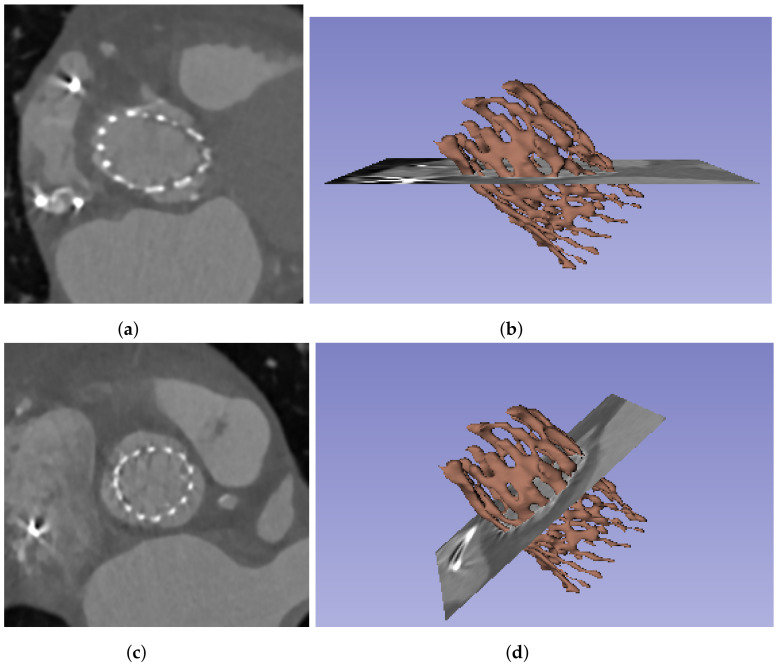
Original and orthogonal views of the CT images captured from a patient with an Allegra of 27 mm implanted. (**a**) Axial view of the original image slices, (**b**) prosthesis 3D reconstruction, with the axial view of the original images, (**c**) orthogonal view of the prosthesis, after the image transformation, (**d**) prosthesis 3D reconstruction, with a plane orthogonal to the prosthesis principal axis.

Several measurements that could be interesting for the characterization of the prosthesis and the implant (meaning the relationship between the device and the patient’s anatomy) are obtained by analyzing the cross-section of the prosthesis and the aortic root, along the principal axis. For such analysis, the measurements must be extracted from planes orthogonal to the prosthesis. An example of an axial slice of the original CT study can be seen in Figure 2a and the 3D reconstruction of the prosthesis in Figure 2b; the prosthesis lies oblique to the three main planes of the CT studies (axial, sagittal, and coronal), and thus the orthogonality is not present. Therefore, it is necessary to transform each CT time volume to obtain the required orthogonality, which makes it possible to extract orthogonal views of the implant.

To perform this transformation in a fully automated way, in this work, we propose to first segment the prosthesis frame and then identify its principal axis, which allows the angles of the desired orthogonal projection to be computed. To explore the relationship between the device and the patient’s anatomy, the patient’s aortic root is also segmented, making it possible to take measurements of the implant, as will be explained in detail. In addition, as we are dealing with 4D data, such measurements could be computed over the time volumes, permitting an analysis over different stages of the cardiac cycle, which could provide valuable information of the assessment of TAVI results. Furthermore, given a cohort of patients, such measurements could also be studied for groups of patients, grouped by the prosthesis model they have been implanted, exploring possible differences among them.

Figure 1 provides a schema of the methodology, which will be explained in more detail in this section. To implement the whole pipeline described in Figure 1, we have developed a set of tools to produce the intermediate processing stages, namely (1) prosthesis segmentation, for identifying the device in each time volume; (2) orthogonal plane extraction, for obtaining the required views over the prosthesis principal axis; and (3) aortic root segmentation, for studying the relationship between the device and the anatomy. This method allows different applications, and some of them are proposed and described in the next sections.

#### 2.3.1. Prosthesis Segmentation

**Figure 3:**
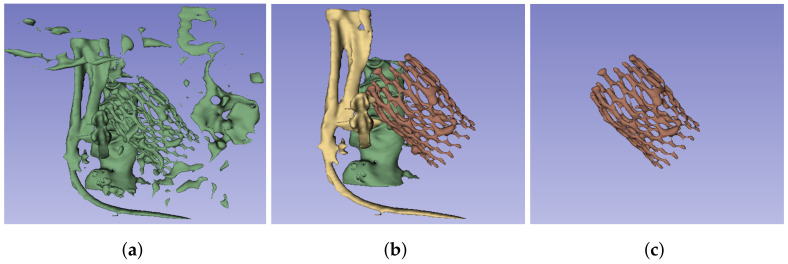
3D reconstruction of the results of the intermediate steps of the prosthesis segmentation process. (**a**) Results after the image thresholding, (**b**) results after the removal of the small islands in the thresholding, (**c**) final prosthesis segmentation, after removing the large structures.

The first step of the pipeline, as depicted in Figure 1, is devoted to the identification and segmentation of the TAVI device in the CT study. The segmentation of the prosthesis consists of three automatic steps: image thresholding, artifact removal, and identification of the prosthesis. For the first step, we propose to apply a lower threshold ($Th_{1}$) to the whole image study, selecting the voxels with intensity values over the threshold (Figure 3a). In this work, this threshold value has been heuristically selected such that it could assure that the voxels corresponding to the prosthesis in the images have greater values in Hounsfield units (HU) than the threshold. The suitable threshold value may differ if prostheses of other models or materials, or even other imaging protocols, are used, but except for the threshold selection, the rest of the method would be still valid. Figure 3a provides an example of the output of the thresholding stage, and, as can be seen, there are two different types of artifacts that can be produced: small speckles and large, dense, anatomical structures. The second step is devoted to removing the former, small groups of connected voxels from the segmentation by setting a second threshold ($Th_{2}$) to the minimum size of the groups, removing smaller ones from the foreground, as shown in Figure 3b. To deal with the large structures, the last step consists of analyzing the remaining groups of connected pixels to identify the prosthesis among them and remove the others. As the prosthesis is a thread-like, hollow structure, whereas the other structures are solid and dense, the density of all the remaining structures is studied, removing the densest structures, obtaining the prosthesis segment as a result.

#### 2.3.2. Orthogonal Plane Extraction

As previously explained, due to the position of the prosthesis, oblique to the three main axes of the CT study, it is necessary to obtain orthogonal planes before extracting the measurements. Before performing such transformation, it is necessary to know the angles generated by the prosthesis principal axis with respect to the coordinate axes. This principal axis can be identified by filling the prosthesis segment, obtaining a solid, cylinder-like structure that can be used to extract the axis direction.

**Figure 4:**
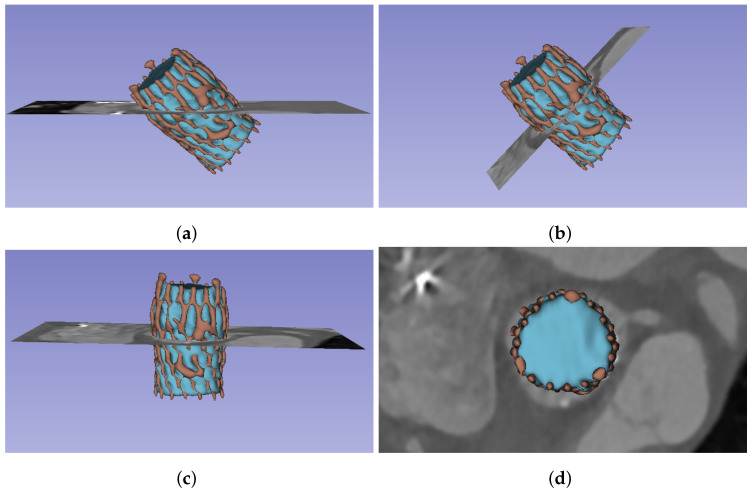
Reformat of the images to obtain orthogonal views along the prosthesis long axis. The images include the 3D reconstruction of the prosthesis frame segment (red) and the filling segment (blue), as well as the planes. (**a**) Original axial views; (**b**) orthogonal plane, obtained after the reformatting; (**c**) orthogonal plane, but rotated, aligning the prosthesis long axis with Y axis; (**d**) orthogonal plane view from the top.

Therefore, the tasks involved in this stage are as follows: fill the prosthesis, identify the principal axis, and extract the orthogonal planes. The first step, filling the prosthesis, consists of identifying the largest internal cavity within the prosthesis segment, obtained in the previous stage. In this work, the Wrap and Solidify algorithm [18] was used, an iterative method that repeats a shrink-wrapping process. After this step, a new solid segment (i.e., filling segment) inside the prosthesis was obtained, as shown in Figure 4a. The second step is devoted to analyzing the new filling segment, allowing the identification of the prosthesis principal axis, defined by its director vector $\vec{v}\left(x,y,z\right)$. The third step consists of extracting the orthogonal planes. This task can be accomplished by computing the angle θ between the director vector of the prosthesis principal axis and the *z* axis ($\vec{z}\left(x,y,z\right)=\left[0,0,1\right]$) using Equation (1) and obtaining the vector normal to both axes ($\vec{n}\left(x,y,z\right)$) using Equation (2). Finally, we can compute the transformation matrix to rotate the images about the center of the study, in the direction of $\vec{n}$ and by θ.

(1) $$\theta =\cos^{-1}\left(\frac{\vec{v}\vec{z}}{|\vec{v}\left|\right|\vec{z}|}\right)$$

(2) $$\vec{n}=\vec{v}\times \vec{z}$$

After applying this transformation, the orthogonal planes are obtained, as can be seen in Figure 4, which will make it possible to take the cross-sectional measurements of the prosthesis and the aortic root.

#### 2.3.3. Aortic Root Segmentation

**Figure 5:**
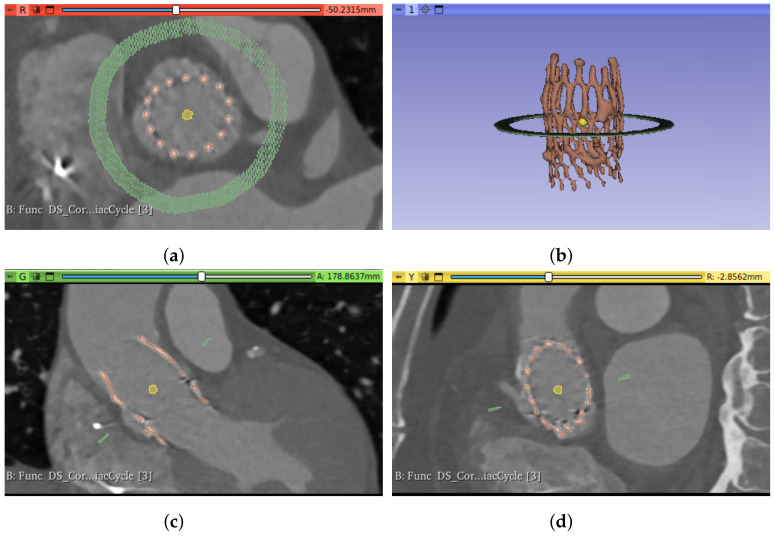
Different views of the prosthesis segmentation (red) and the seeds used to initialize the GrowCut algorithm for the aortic root segmentation: foreground seed (yellow) and background seed (green). (**a**) Axial view, (**b**) 3D reconstruction, (**c**) sagittal view, (**d**) coronal view.

In addition to identifying the prosthesis, this method requires the extraction of the aortic root, which will allow the implant characterization to be obtained. In this work, the GrowCut algorithm [19] was used for the aortic root segmentation. The initialization of this algorithm requires the selection of two seeds: one for the foreground (meaning the object to segment, in this case, the aortic root lumen) and the other for the background (the rest of the image). For the automatic selection of the seeds, we propose to use a sphere of radius $R_{1}$ centered in the centroid of the filling segment for the foreground seed. For the background seed, we propose to use a circumference of radius $R_{2}$ centered on the center of mass of the prosthesis on a plane orthogonal to the principal axis and at the height of the center of mass; see Figure 5 to observe both seeds.

**Figure 6:**
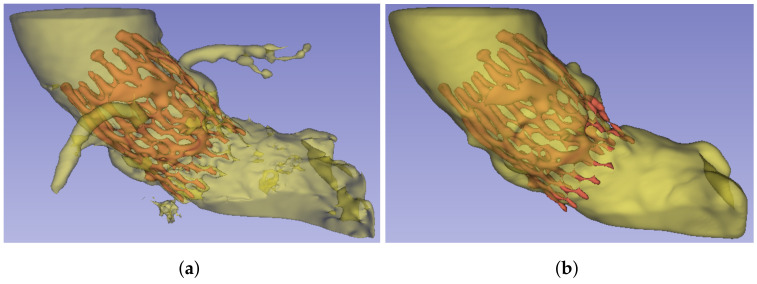
Aortic root segmentation. (**a**) Segmentation obtained by the GrowCut algorithm, using the seeds defined in Figure 5, (**b**) post-processed version of the aortic root segmentation after removing noise and the coronary arteries as well as flattening the surface.

The algorithm starts from the selected seeds and builds a graph, whose edge weights are related to the pixel intensity difference in between, and then determines the shortest distance in such weights using a version of the Dijkstra algorithm [19]. Figure 6a shows an example of the result of the algorithm. This segmentation is further post-processed to remove the noise, smooth the external surface, and remove the coronary arteries, as they are not required for the subsequent analyses proposed in this work. For this purpose, a copy of the segment is shrunk after growth, and the result is used to mask the first aortic root segmentation, removing the noisy artifacts that were present in the GrowCut segmentation. Finally, the segmentation surface is smoothed using the median filter. Figure 6b provides an example of the result of this aortic root segmentation stage.

### 2.4. Applications

The previously explained method can be employed to extract clinical information in post-TAVI scenarios. Some of the possible applications are presented in this work, classified in two different groups, namely the prosthesis characterization, which refers to measurements of the device itself, and the implant characterization, which describes the relationship between the device and the patient anatomy, meaning the aortic root.

#### 2.4.1. Prosthesis Characterization

The aim of this application is to automatically extract parameters of the frame itself from the 4D-CT, making it possible to analyze its behavior over the cardiac cycle. Among all the metrics that can be extracted from the images and that can characterize the prosthesis, a possible classification is to distinguish the following three categories: (1) metrics that characterize the prosthesis as a 3D object, such as the volume, the center of mass, or the angles defined by the principal axis; (2) metrics that characterize the prosthesis by analyzing the 2D cross-sections over the principal axis, such as the CSA, perimeter of the cross-section, or the maximum diameter of the cross-section; and (3) metrics that characterize the evolution over the cardiac cycle.

Concerning the first group, the metrics derived from the 3D object can be directly extracted from the frame segmentation, i.e., the result of Section 2.3.1, as they do not require orthogonality. On the other hand, cross-sectional metrics must be extracted from orthogonal planes, requiring the step in Section 2.3.2. Once the orthogonal planes are obtained, the prosthesis 3D segmentation is sliced along its principal axis, obtaining a stack of 2D images.

**Figure 7:**
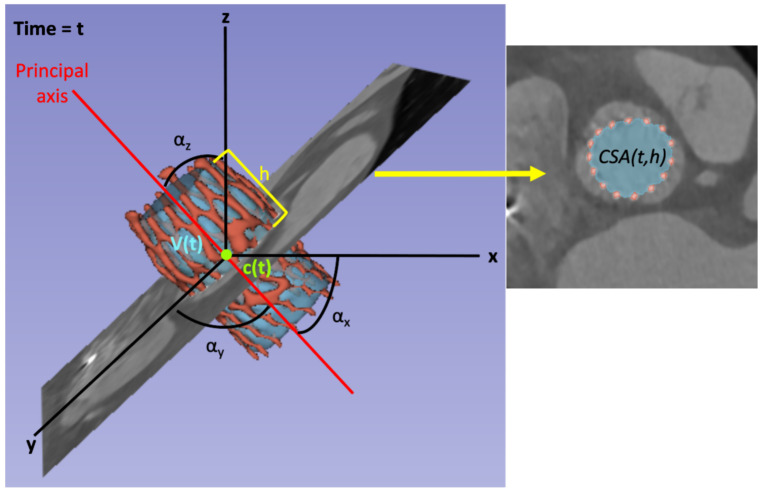
Schema of the prosthesis parameters studied in this work for a given time instant *t*. On the left, the figure shows the 3D reconstruction of the TAVI frame (red) and its filling (blue) segments, along with the coordinate axes [x,y,z], the prosthesis principal axis (red line), and an example of a region of interest (ROI) of an orthogonal plane. The variables V(t) and c(t) refer to the prosthesis volume and center of mass in instant *t*, respectively. The variables $\alpha_{x}$, $\alpha_{y}$, and $\alpha_{z}$ represent the angles between the principal axis and the coordinate axes. The orthogonal plane located at height *h*, shown in the 3D view, is also displayed in the right part of the figure. The 2D view on the right shows the cross-sections of the segments at *h* and the parameter CSA(t,h), which refers to the CSA of the filling segment at time instant *t* and height *h*.

All the metrics can be studied over the cardiac cycle, e.g., the prosthesis volume V(t), where *t* determines the time instant; and cross-sectional metrics can also be analyzed along the principal axis, e.g., the cross-sectional area CSA(t,h), where *t* determines the time instant and *h* determines the location of the orthogonal plane with respect to the principal axis. In this work, *h* is defined as the distance between the tip of the prosthesis closer to the sinotubular junction and the location of the plane in the direction of the principal axis, measured as a percentage of the length of the prosthesis long axis. Therefore, the plane at the prosthesis outflow would correspond with h=0%, the plane at the prosthesis inflow (i.e., the tip closer to the annulus) would be h=100%, and all the intermediate locations would range between these values. See Figure 7 for a graphical schema of the metrics considered in this work. The variable V(t) refers to the inner volume of the prosthesis for the time instant *t*. The variables $c\left(t\right)=\left[c_{x},c_{y},c_{z}\right]$ and $\alpha \left(t\right)=\left[\alpha_{x},\alpha_{y},\alpha_{z}\right]$ correspond to the center of mass of the prosthesis and the angle between the principal axis and the axes of coordinates for the time instant *t*, respectively, and they have three components, one for each axis.

**Figure 8:**
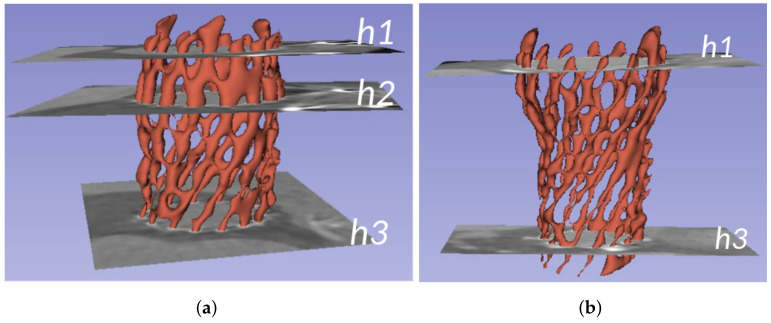
Example of the 3D reconstruction of different prosthesis models with the specific planes $h_{1}$, $h_{2}$, and $h_{3}$, defined for the prosthesis characterization. (**a**) Allegra 27 mm, (**b**) CoreValve 26 mm ($h_{2}$ coincides with $h_{1}$).

For characterizing the prosthesis, three specific planes have been defined for a deeper analysis. These planes are $h_{1}$, located at the prosthesis outflow; $h_{2}$, located at the maximum diameter of the prosthesis; and $h_{3}$, located at the prosthesis inflow. Figure 8a displays these planes for an Allegra 27 mm prosthesis. In the case of the CoreValve Evolut R, the maximum diameter of the prosthesis is located at the prosthesis outflow, meaning that $h_{1}$ and $h_{2}$ would be placed at the same height (see Figure 8b).

#### 2.4.2. Implant Characterization

While the prosthesis characterization, previously described, is centered on the device itself and its movement, the implant characterization seeks to depict the prosthesis’s position within the patient’s aortic root and its changes throughout the cardiac cycle and at different levels along the prosthesis axis. Consequently, this application requires the identification of two structures within the CT scan: the prosthesis and the aortic root, segmented using the described method. Subsequently, orthogonal planes along the principal axis of the prosthesis are extracted, enabling the examination of cross-sections for both structures.

**Figure 9:**
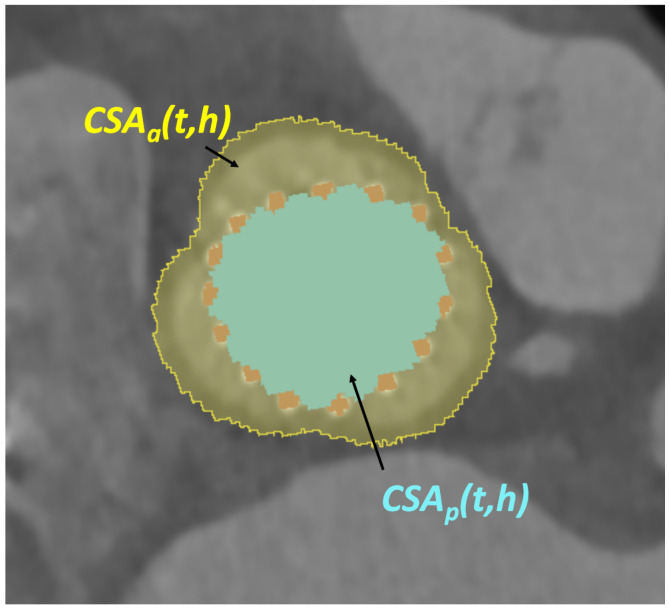
Cross-section of the TAVI frame and the aortic root segmentations, orthogonal to the prosthesis axis. The CSAs of the prosthesis (blue) and the aortic root (yellow) are defined for a given plane *h* and time instant *t*.

To facilitate this, the terms $CSA_{a}\left(t,h\right)$ and $CSA_{p}\left(t,h\right)$, which are visually depicted in Figure 9, are introduced. The term $CSA_{a}\left(t,h\right)$ represents the CSA of the aortic root at the specified height *h* and time *t*. Similarly, $CSA_{p}\left(t,h\right)$ pertains to the CSA of the prosthesis at the same height and time instant. Furthermore, dCSA(t,h) is defined as the difference between the CSA of the aorta and that of the prosthesis at such a height and time instant. Analyzing the values of this newly introduced variable, dCSA(t,h), allows us to examine the anchoring of the prosthesis within the patient’s aortic root at various levels and observe its changes over time. For instance, at the prosthesis inflow level (closer to the annulus), a lower value of dCSA can be expected, as it is where the device is fixed to the patient’s anatomy. In contrast, at the sinus level, as depicted in Figure 9, dCSA attains higher values. The analysis of the variation of these three variables along the axis and over time enables the investigation of the biomechanical interaction between the device and the patient’s anatomical structures, which can be related to potential complications, such as paravalvular regurgitation, patient–prosthesis mismatch, prosthesis malposition, or abnormal expansion of the prosthesis.

## 3. Results

In order to evaluate the presented method, it has been implemented in Python 3.9.10 and using 3D Slicer 5.2.2, and it has been tested with the dataset described in Section 2.1.

Regarding the parameters defined in the method, we used $Th_{1}=800$ HU and $Th_{2}=11,000$ voxels. Some tasks in the method were implemented using 3D Slicer modules, namely SurfaceWrapSolidify [18] (“Largest cavity” mode for six iterations) to fill the prosthesis segment, SegmentStatistics to obtain metrics from the generated segments, and SegmentGeometry [20] to extract the orthogonal plane and the cross-sections. Concerning the aortic root segmentation, the “Grow from seeds” algorithm 3D Slicer’s implementation was used (for one iteration and “Seed locality” set to 0). The algorithm initialization was as described in Section 2.3.3, with $R_{1}=2$ voxels and $R_{2}=40$ voxels. Supplementary Videos S1 and S2 provide animations of a sweep over the orthogonal cross-sections of the prosthesis along its principal axis for a specific time stamp of a patient (Allegra 27 mm device). Cross-sections were automatically obtained using the method described in this work. Supplementary Video S2 also shows the obtained segmentations, namely the prosthesis (red), its filling (blue), and the aortic root (yellow).

Several experiments were conducted to test the proposed method, addressing the presented applications and using the previously described dataset. The first application is devoted to the prosthesis characterization, the second to the implant characterization, and the third to the characterization of the whole cohort, analyzing features studied in the previous applications but grouping the patients by their prosthesis model. The purpose of these experiments lies in proving the validity of the method; note that a wider dataset would be required to extract clinical conclusions.

### 3.1. Prosthesis Characterization

For prosthesis characterization, the parameters shown in Figure 7, namely the prosthesis volume V(t), the center of mass c(t), and the CSAs along the axis CSA(t,h), have been automatically extracted for the whole cohort of patients using the presented method, allowing their study over the cardiac cycle.

**Figure 10:**
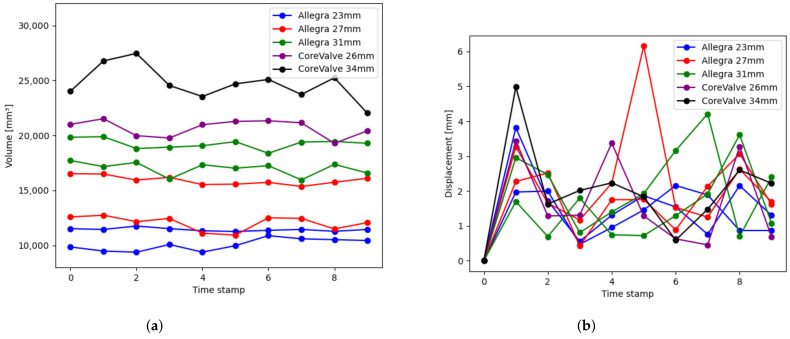
Results obtained for the prosthesis characterization as a 3D object for eight patients with different prosthesis models (Allegra 23 mm in blue, Allegra 27 mm in red, Allegra 31 mm in green, CoreValve 26 mm in purple, CoreValve 34 mm in black). (**a**) Evolution of the inner volume of the prosthesis over the cardiac cycle for the patients with the minimum and maximum mean volume (computed over all the time volumes) for each prosthesis model, (**b**) Displacement of the prosthesis center of mass over the cardiac cycle for the patients with the minimum and maximum mean displacement (computed over all the time volumes) for each prosthesis model.

Figure 10a shows the evolution of the internal volume of the prosthesis over the cardiac cycle for eight patients, two patients with each Allegra size (23 mm, 27 mm, and 31 mm) and two patients with CoreValve (26 mm and 34 mm). These patients were selected by choosing the patients in the dataset with minimum and maximum mean volume for each prosthesis model. This experiment reveals that the volume curves for each patient slightly oscillate over a mean value, with values that range from 10,069.28 mm^3^ for Allegra 23 mm to 24,706.10 mm^3^ for CoreValve 34 mm. CoreValve patients have greater mean volume values than Allegra ones. Analyzing both manufacturers separately, it can be seen that the prosthesis inner volume grows with the nominal size of the prosthesis model. Notice that CoreValve 26 mm has greater volume values than Allegra 31 mm, even though the nominal size is lower, but this is due to the differences in the shapes of each model. Regarding the volume maximum variation over the cardiac cycle, CoreValve patients have also shown greater variations (5430.89 mm^3^ for CoreValve 34 mm versus 495.81 mm^3^ for Allegra 23 mm).

The displacement of the prosthesis, which represents the distance covered by the prosthesis between two consecutive time stamps, can be calculated throughout the cardiac cycle as detailed in Equation (3). Specifically, the displacement at time stamp *t*, denoted as d(t), is determined by the magnitude of $\vec{u}\left(t\right)$, a vector calculated between the coordinates of the prosthesis’ centers of mass ($c\left(t\right)=[c_{x}\left(t\right),c_{y}\left(t\right),c_{z}\left(t\right)])$ at time stamp *t* and the preceding time stamp, t−1.

(3) $$d\left(t\right)=|\vec{u}|,\;\;\mathrm{where}\;\;\vec{u}=\left[c_{x}\left(t\right)-c_{x}\left(t-1\right),\;c_{y}\left(t\right)-c_{y}\left(t-1\right),\;c_{z}\left(t\right)-c_{z}\left(t-1\right)\right].$$

Figure 10b provides the displacement of the prosthesis center of mass over the cardiac cycle for those eight patients with five different prosthesis models. As in the previous volume figure, the patients displayed in Figure 10b are those with the maximum and minimum mean displacement for each device model, ranging from 2.70 mm to 6.15 mm. There is no perceivable impact of the prosthesis model or size on the displacement, which can be explained by the fact that the movements of the prosthesis are due to heart beating, and the movement depends on the patient’s anatomy, regardless of the device model.

**Figure 11:**
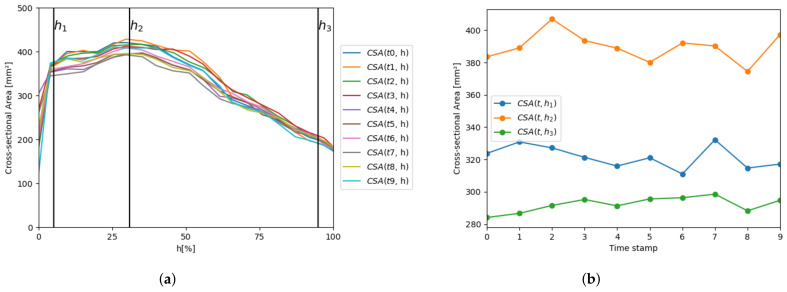

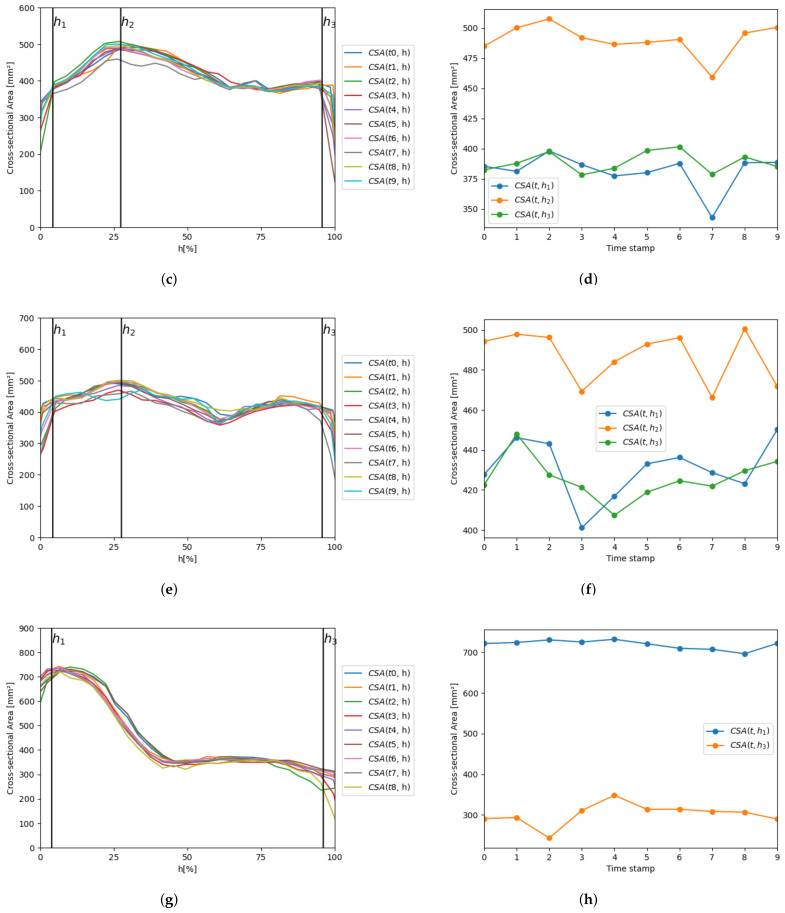
Analysis of the CSA of the prosthesis along the principal axis and over the cardiac cycle for four patients, with different prosthesis models implanted. (**a**) CSA evolution along the principal axis for a patient with an Allegra 23 mm, where each curve corresponds to a different time stamp, besides three vertical lines determining the planes $h_{1}$, $h_{2}$, and $h_{3}$; (**b**) CSA evolution over the cardiac cycle for the same patient in (**a**), for the three planes $h_{1}$, $h_{2}$, and $h_{3}$. The next pairs of graphs replicate the same analysis for patients with other prosthesis models: (**c**,**d**) Allegra 27 mm, (**e**,**f**) Allegra 31 mm, and (**g**,**h**) CoreValve 26 mm.

The next experiments analyze the prosthesis cross-sections along the principal axis for different prosthesis models. Figure 11a shows the evolution of the CSA along the axis for a single patient with Allegra 23 mm implanted, where each curve corresponds to a different time stamp. Figure 11b displays the CSA evolution at the specific planes of interest ($h_{1}$, $h_{2}$, and $h_{3}$, depicted in Figure 8), allowing a deeper analysis of the prosthesis behavior in such planes. Figure 11c–h replicate the same information for other patients, with different device models. The next experiments analyze the prosthesis cross-sections along the principal axis for different prosthesis models. Figure 11a shows the evolution of the CSA along the axis for a single patient with Allegra 23 mm implanted, where each curve corresponds to a different time stamp. Figure 11b displays the CSA evolution over the cardiac cycle at the specific planes of interest ($h_{1}$, $h_{2}$, and $h_{3}$, depicted in Figure 8), allowing a deeper analysis of the prosthesis behavior in such planes. Figure 11c–h replicate the same information for other patients, with different device models.

For each device model, all the curves exhibit the same behavior, with variations over different phases of the cardiac cycle, due to the relaxation and contraction of the heart. The particular shape of each patient’s CSA is characteristic of each prosthesis model, but it is also determined by the patient’s anatomy. For these specific patients, it can be observed that CSA behavior is similar from $h_{1}$ to $h_{2}$, as its value increases, whereas there are more differences from $h_{2}$ to $h_{3}$; CSA decreases almost linearly for Allegra 23 mm (Figure 11a), it presents a decreasing phase (until around *h* = 63%) followed by a flat phase for Allegra 27 mm (Figure 11c), and it displays a local minimum at *h* = 63% and a local maximum at *h* = 80% for Allegra 31 mm (Figure 11e). Concerning the CoreValve (Figure 11g), there are significant differences in the shape, with the maximum achieved at $h_{1}$, decreasing until *h* = 40%, followed by a flatter phase until $h_{3}$. As observed in the volume curves plotted in Figure 11a, the CSA values increase with the device nominal size, with average values of 389.65 mm^3^ for Allegra 23 mm, 486.92 mm^3^ for Allegra 27 mm, and 490.51 mm^3^ for Allegra 31 mm at $h_{2}$; and 702.80 mm^3^ for CoreValve 26 mm at $h_{1}$. Focusing on the planes $h_{1}$ and $h_{3}$ for Allegra models, both tips of the device, for Allegra 23 mm, the minimum values are obtained at $h_{3}$ (Figure 11b), whereas for the models of 27 mm (Figure 11d) and 31 mm (Figure 11f), the CSA values at $h_{1}$ and $h_{3}$ are closer (averages of 381.67 mm^2^ at $h_{1}$ and 388.72 mm^2^ at $h_{3}$ for Allegra 27 mm and 430.60 mm^2^ at $h_{1}$ and 425.58 mm^2^ at $h_{3}$ for Allegra 31 mm).

### 3.2. Implant Characterization

**Figure 12:**
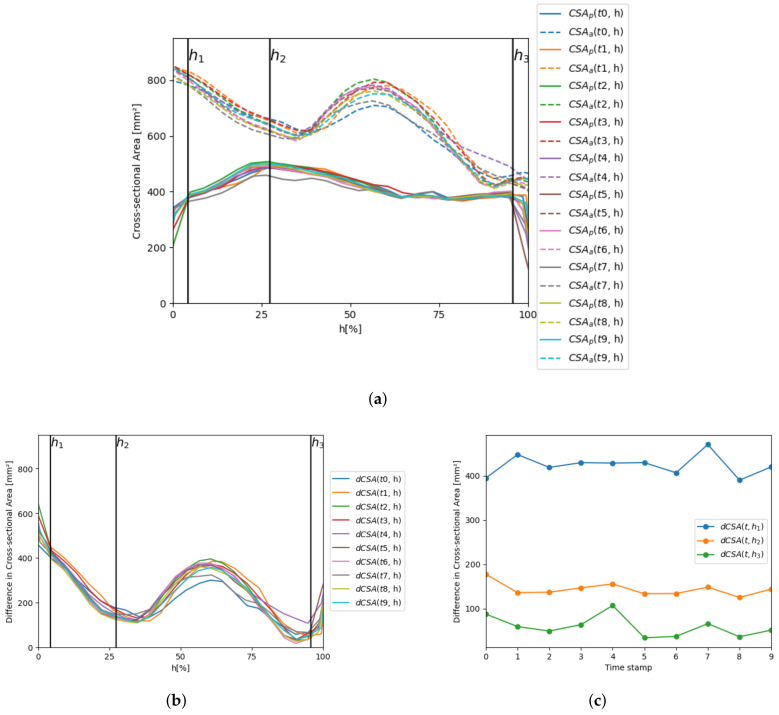
CSAs of the prosthesis and the aorta and dCSA for a single patient (Allegra 27 mm), besides the planes $h_{1}$, $h_{2}$, and $h_{3}$. (**a**) $CSA_{p}\left(t,h\right)$ and $CSA_{a}\left(t,h\right)$ along the principal axis for each time stamp; (**b**) dCSA(t,h) along the principal axis for each time stamp; (**c**) dCSA(t,h) evolution over the cardiac cycle for each of the specific planes $h_{1}$, $h_{2}$, and $h_{3}$.

In this application, the cross-section analysis has been replicated with the two structures at the same time, extracting $CSA_{p}\left(t,h\right)$ and $CSA_{a}\left(t,h\right)$ parameters. Figure 12a shows the variation of such parameters along the prosthesis principal axis for a single patient (Allegra 27 mm) in each time volume of the 4D-CT sequence. Figure 12b represents the variable dCSA(t,h) along the axis for each time stamp for the same patient as the previous figure, and Figure 12c displays the evolution of dCSA over the cardiac cycle for the planes $h_{1}$, $h_{2}$, and $h_{3}$.

This experiment can study the anchoring of the prosthesis to the patient’s anatomy. As can be seen in Figure 12b, $CSA_{a}$ is significantly larger than $CSA_{p}$ at $h_{1}$ (average dCSA of 423.80 ± 22.69 mm^2^). Then, the difference progressively decreases (average dCSA of 143.99 ± 14.01 mm^2^ at $h_{2}$) until reaching a local minimum at *h* = 30% (average dCSA of 122.27 ± 13.18, mm^2^). Afterwards, the difference increases, reaching a local maximum at the level of the sinuses at *h* = 55% (average dCSA of 359.94 ± 27.07 mm^2^). Finally, the difference drops, and the minimum difference is obtained at the annulus (average dCSA of 59.41 ± 22.23 mm^2^ at $h_{3}$), where the prosthesis is anchored to the aorta. In Figure 12c, it can be observed that dCSA has smooth variations over the cardiac cycle in the three planes, showing the least variation at $h_{2}$ (difference between the maximum and minimum values of dCSA in the 4D-CT sequence of 80.36 mm^2^, 52.43 mm^2^, and 72.89 mm^2^, at $h_{1}$, $h_{2}$, and $h_{3}$, respectively). The same analysis has been conducted in all the patients in the cohort, and results on average for each device model are provided in the following section.

### 3.3. Cohort Characterization

The previous applications, when performed in the whole cohort, can be utilized to study the patients grouped by the prosthesis model they have been implanted, allowing exploration of the differences among the parameters from each of the prosthesis sizes. In this experiment, the five device models available in the dataset have been separately analyzed; however, note that the dataset size is not large enough to extract general conclusions.

Table 1 gathers the results of different metrics, which will be described in this section, computed in mean and standard deviation separately for all the patients with the same device model. Each column provides the results for each prosthesis model and size. Regarding the rows, the first specifies the device size and the second row the number of patients used in the computations for each model. The third row, $V_{m}$, refers to the mean inner volume of the prosthesis in the whole 4D-CT sequence, and the forth row, ΔV, refers to the difference between the maximum and minimum volume values in each sequence. In the same way, the following rows represent the same metrics, mean and maximum difference over the cardiac cycle, for the displacement (Equation (3)). The seventh row contains the mean CSA of the prosthesis at $h_{1}$ over the 4D-CT sequence and the eighth the difference between the maximum and the minimum CSA values at $h_{1}$. The next two rows replicate the same computations for dCSA. The remaining rows in the table repeat the computations from 7th to 10th rows with the other planes, namely $h_{2}$ and $h_{3}$.

Analyzing the results in Table 1, it can be observed that both $V_{m}$ and ΔV increase with the size of the prosthesis for each of the manufacturers ($V_{m}$ from 10,798.20 mm^3^ to 18,172.35 mm^3^ for Allegra 23 and 31 mm, respectively, and from 20,668.97 mm^3^ to 24,706.10 mm^3^ for CoreValve 26 and 34 mm, respectively). As explained in the prosthesis characterization (Section 3.1) and illustrated in Figure 10a, this trend was anticipated, given the evident relationship between the prosthesis nominal size and its inner volume. Additionally, variations between manufacturers are expected due to differences in the prosthesis design. Regarding the displacement, there is no perceivable trend in its mean with the device model. The obtained $d_{m}$ values range from 1.36 mm to 1.96 mm, with significantly high values in the average maximum variation, Δd, with values from 2.90 mm to 4.98 mm. These results agree with the behavior observed in Figure 10b.

Regarding the CSA results, the minimum $CSA_{m}$ for Allegra 23 mm is obtained at $h_{3}$ (240.12 mm^2^), i.e., at the frame inflow, whereas for the other Allegra sizes, it is achieved at $h_{1}$ (348.47 mm^2^ for 27 mm and 407.82 mm^2^ for 31 mm). The maximum $CSA_{m}$, by definition located at $h_{2}$ for Allegra devices, grows with the prosthesis’ nominal size (from 396.35 mm^2^ for 23 mm to 485.34 mm^2^ for 31 mm). This relationship is attributed to the direct correlation between the prosthesis’ maximum diameter and its nominal size. In the case of CoreValve models, $CSA_{m}$ also grows with the device’s nominal size, reaching the maximum value at $h_{1}$ (718.79 mm^2^ for 26 mm and 803.42 mm^2^ for 34 mm). Both models exhibit notable disparity between the CSA values at $h_{1}$ and $h_{3}$ (303.41 mm^2^ for 26 mm and 540.37 mm^2^ for 34 mm). However, it is worth noting that this difference is more pronounced for the CoreValve 26 mm, given the distinctive hourglass shape of the CoreValve 34 mm. Additionally, it is important to highlight that $CSA{\left(h_{2}\right)}_{m}$ is unavailable for CoreValve models since $h_{2}$ has not been defined for these device models. This is because the plane of maximum diameter coincides with the same position as $h_{1}$. Consequently, $\Delta CSA(h_{2})$, $dCSA{\left(h_{2}\right)}_{m}$ and $\Delta dCSA(h_{2})$ are also not applicable to CoreValve devices for the same reason.

Concerning ΔCSA, it tends to grow with the prosthesis size at all the analyzed planes for Allegra devices (46.98–83.91 mm^2^ at $h_{1}$, 35.77–57.61 mm^2^ at $h_{2}$, 30.32–48.47 mm^2^ at $h_{3}$), and the minimum ΔCSA is obtained at $h_{3}$ for all the Allegra models. Observing the ΔCSA results obtained for all the device models, it is remarkable that CoreValve devices obtain lower values at $h_{1}$ than Allegra ones (46.98–83.91 mm^2^ for Allegra, 35.52–40.92 mm^2^ for CoreValve). A possible explanation for this fact is that the stent frame of Allegra devices is flexible, permitting stent tip deflection, which allows the commissural point of the leaflets to move with every cardiac cycle, whereas CoreValve Evolut R does not present this feature in its design. Therefore, more variations at $h_{1}$ were expected for Allegra devices.

Regarding $dCSA_{m}$, the greatest values for Allegra devices are obtained at $h_{1}$ (366.51–606.73 mm^2^ for 23 mm and 31 mm, respectively), and the lowest at $h_{3}$. This phenomenon is attributed to the prosthesis being anchored to the patient’s aortic root at the annulus. In contrast, the frame outflow exhibits greater freedom of movement, resulting in a larger difference between the CSA of the aorta and that of the prosthesis. At $h_{1}$ and $h_{2}$, $dCSA_{m}$ tends to increase with the device size, whereas $dCSA{\left(h_{3}\right)}_{m}$ decreases as the device size increases (184.40–39.98 mm^2^ for 23 mm and 31 mm, respectively, at $h_{3}$). The same behavior is observed for ΔdCSA; it also grows with the size at $h_{1}$ and $h_{2}$ (71.66–135.24 mm^2^ for 23 mm and 31 mm, respectively) and the opposite way for $h_{3}$ (116.25–49.73 mm^2^ for 23 mm and 31 mm, respectively). Concerning CoreValve patients, $dCSA{\left(h_{1}\right)}_{m}$ decreases with the prosthesis size (246.70 mm^2^ for 26 mm and 160.50 mm^2^ for 34 mm), and $dCSA{\left(h_{3}\right)}_{m}$ increases (115.41 mm^2^ for 26 mm and 265.95 mm^2^ for 34 mm). As observed in $\Delta CSA(h_{1})$, $\Delta dCSA(h_{1})$ is considerably lower for CoreValve cases than for Allegra, which could also be explained by the flexibility of Allegra stent frames.

## 4. Discussion

This work presents a method for the automatic extraction of information from 4D-CT post-TAVI images, proposing a selection of tools for its implementation. The method has been tested using images from patients with specific prosthesis models (Allegra 23, 27, and 31 mm and CoreValve 26 and 34 mm), but it could work using different devices with limited changes. Nitinol-based auto-expandable prostheses should not require modifications, whereas chromo-cobalt balloon-expendable ones could require adjustment of the threshold value associated with the value in HU of each alloy metallic attenuation. As commented when presenting the method, one task that would vary for different devices is the value of the threshold values used in the prosthesis frame segmentation, $Th_{1}$ and $Th_{2}$. In this work, the values have been heuristically set; however, it would be convenient to explore other automatic strategies for setting these values in order to make the method more robust. The selection of the threshold values directly affects the prosthesis segmentation on which the remainder of the method relies; therefore, it is a key task. More sophisticated segmentation methods, for instance taking into account the prior knowledge of the prosthesis structure or using deep learning, would help to improve the segmentation accuracy, possibly improving the results of the rest of the tasks.

Regarding the aortic root segmentation, there are multiple works in the literature [21,22] for the automatic segmentation of heart structures and, more specifically, the aorta. However, it is not as common to segment the aortic root with a prosthesis implanted, which is the case in this work. Post-TAVI images involve a higher level of difficulty for the segmentation of the aorta, as the device generates artifacts in the images. As previously discussed for the prosthesis segmentation, exploring other tools for the aorta segmentation could improve the method robustness and accuracy. The method requires the automatic determination of two seeds; the foreground seed is obtained automatically using the mass center of the prosthesis previously determined, and the foreground seed uses a circular sector of radius $R_{2}$, heuristically set to 40 voxels, that allows the aortic root to be left inside. In cases of extremely large roots or a scanning protocol that enlarges the heart, this parameter could be different and could require a modification in order to guarantee that the aortic root lies within the automatically generated surface and thus the background seed is valid.

The method has been tested on a cohort of 13 patients, and the average results are provided in Table 1. The main conclusions extracted from the results are that both $V_{m}$ and ΔV increased with prosthesis size across Allegra and CoreValve devices, reflecting their nominal size correlation. Displacement ($d_{m}$) showed no distinct trend. In terms of CSA, differences were observed based on nominal size and measurement plane for both Allegra and CoreValve. Regarding the variation of CSA (ΔCSA), it tends to increase with prosthesis size for Allegra devices at all analyzed planes, with the minimum ΔCSA consistently observed at $h_{3}$. Interestingly, CoreValve devices display lower ΔCSA values at $h_{1}$ compared to Allegra devices, potentially attributable to stent tip deflection in Allegra devices. As for $dCSA_{m}$, the highest values for Allegra devices are found at $h_{1}$, while the lowest values occur at $h_{3}$ due to the prosthesis’s anchoring at the patient’s aortic root. $dCSA{\left(h_{1}\right)}_{m}$ and $dCSA{\left(h_{2}\right)}_{m}$ generally increase with device size, whereas $dCSA{\left(h_{3}\right)}_{m}$ decreases. The same trends are observed for ΔdCSA. For CoreValve patients, $dCSA{\left(h_{1}\right)}_{m}$ decreases with prosthesis size, while $dCSA{\left(h_{3}\right)}_{m}$ increases. Overall, these findings offer valuable insights into prosthesis behavior, encompassing size-related variations and inter-model differences.

While this patient cohort serves to test the proposed method, more general insights could be extracted from a larger dataset, specially for CoreValve devices (or other manufacturers). Such CoreValve patients were included in this study to demonstrate that the present method can successfully be used for the analysis of TAVI prosthesis with different shape characteristics. It would also be interesting to perform a longitudinal study, acquiring 4D-CT images from the same patients, months or years after, and analyzing them to determine whether there are meaningful differences. In addition, besides the metrics considered in the results section, the method can extract many other features from the images, such as the inclination of the prosthesis axis, the maximum diameter, and the eccentricity of the cross-sections. Different planes could also be studied as well, given that the method can extract any orthogonal plane.

One consideration that would be interesting to take into account when analyzing the evolution of the cross-section of a specific plane over the cardiac cycle is the fact that the contraction movements of the prosthesis, produced by the heart beating, may have a component in the direction of the principal axis direction. This makes the length of the prosthesis vary over the 4D-CT sequence, and therefore a specific plane at a specific height *h* would not correspond to the same location in a different time stamp. In this work, the variations of the prosthesis length in the axis direction over the time volumes have been considered negligible; however, it would be opportune to track landmarks over the sequence for an accurate identification of the plane at each time stamp.

The primary objective of this research is to develop an automated method capable of extracting relevant information from post-TAVI 4D-CT scans while also demonstrating its applicability across various prosthesis models. In order to establish the reliability of this method, it is essential to test it using a more extensive dataset, including patients with a wider range of device models and even from other hospitals. The challenge in this validation process lies in the absence of established, state-of-the-art tools that can yield comparable measurements. As an alternative strategy, we propose a manual approach involving the segmentation of both the prosthesis and aortic root, followed by the manual extraction of all the measurements considered in this study, including the prosthesis inner volume, its center of mass, and the CSA of the prosthesis and the aorta. Ideally, this validation should involve external experts in the field to ensure objectivity. Nevertheless, it is important to acknowledge that this manual process becomes unfeasible when dealing with a substantial dataset due to the sheer volume of data involved. Furthermore, it is also pertinent to remark that the results derived from this method in our cohort of patients have been analyzed by the authors, which include cardiologists and prosthesis specialists. Their analysis confirms the logical alignment of the results with related factors such as prosthesis design and patient-specific characteristics.

Regarding the clinical interest of the method, the proposed characterization may be useful for the assessment of the biomechanical behavior of the prosthesis and the expected long-term damage models, as well as the thrombosis and the valve alignment. A long-term study with a larger volume of patients would provide the statistical robustness necessary to include these metrics within the clinical procedure, but this falls out of the scope of this paper.

## 5. Conclusions

This work presents a new method for the automatic characterization of TAVI results in 4D-CT imaging. The fully automated method uses an image processing and artificial intelligence pipeline consisting of prosthesis frame segmentation, extraction of orthogonal planes, and aortic root segmentation. This approach enables applications of clinical interest, including prosthesis, implant, and cohort characterizations, as proposed in this work. The method has been evaluated in a cohort of 13 patients with five different TAVI device models, providing the results of the proposed applications in terms of geometric features computed over the cardiac cycle, characterizing the prosthesis as a 3D object (volume, center of mass) and studying the cross-sections of the aorta and the prosthesis along its axis (CSA and dCSA). The automated extraction of these parameters offers valuable clinical information, promising procedural enhancements in TAVI and substantial reductions in the time and effort required for image interpretation. Nonetheless, it is important to note that a long-term study involving a larger patient cohort would be necessary to establish the statistical robustness required for the integration of these metrics into clinical practice. Such an extensive study, however, is beyond the scope of this paper.

## Figures and Tables

**Table 1:** Results, computed in mean and standard deviation, for the whole cohort of patients, grouped by their implanted device model.

	Allegra			CoreValve	
size (mm)	23	27	31	26	34
*n*	3	5	3	1	1
$V_{m}$ (mm${}^{3})$	10,798.20 ± 564.98	14,039.95 ± 1361.54	18,172.35 ± 920.84	20,668.97	24,706.10
ΔV (mm${}^{3})$	962.21 ± 415.69	1779.51 ± 341.29	2113.74 ± 687.48	2232.64	5430.89
$d_{m}$ (mm)	1.36 ± 0.10	1.82 ± 0.19	1.68 ± 0.26	1.57	1.96
Δd (mm)	2.90 ± 0.69	4.04 ± 1.11	3.67 ± 0.42	3.43	4.98
$CSA{\left(h_{1}\right)}_{m}$ (mm${}^{2})$	346.48 ± 28.54	348.47 ± 32.68	407.82 ± 13.16	718.79	803.42
$\Delta CSA(h_{1})$ (mm${}^{2})$	46.98 ± 10.60	46.15±17.16	83.91 ± 22.97	35.52	40.92
$CSA{\left(h_{2}\right)}_{m}$ (mm${}^{2})$	396.35 ± 9.07	436.85 ± 33.94	485.34 ± 6.26	–	–
$\Delta CSA(h_{2})$ (mm${}^{2})$	35.77 ± 2.08	57.61 ± 9.46	57.41 ± 17.62	–	–
$CSA{\left(h_{3}\right)}_{m}$ (mm${}^{2})$	240.12 ± 48.91	357.07 ± 22.71	466.25 ± 56.00	303.41	540.37
$\Delta CSA(h_{3})$ (mm${}^{2})$	30.32 ± 3.25	37.52±22.44	48.47±18.16	23.03	71.78
$dCSA{\left(h_{1}\right)}_{m}$ (mm${}^{2})$	366.51 ± 76.65	430.98 ± 41.77	606.73 ± 31.03	246.70	160.50
$\Delta dCSA(h_{1})$ (mm${}^{2})$	71.66 ± 4.17	90.96 ± 22.23	135.24 ± 17.40	61.00	63.82
$dCSA{\left(h_{2}\right)}_{m}$ (mm${}^{2})$	188.71±79.81	185.18 ± 51.70	309.47±33.65	–	–
$\Delta dCSA(h_{2})$ (mm${}^{2})$	45.45 ± 14.70	70.96 ± 25.79	129.97 ± 12.98	–	–
$dCSA{\left(h_{3}\right)}_{m}$ (mm${}^{2})$	184.40 ± 162.15	58.67 ± 27.86	39.98 ± 15.01	115.41	265.95
$\Delta dCSA(h_{3})$ (mm${}^{2})$	116.25 ± 56.23	66.12 ± 9.25	49.73 ± 12.25	76.19	108.36

## Data Availability

The data and code that support the findings of this work are available from the corresponding author C.V. upon reasonable request.

## Supplementary Materials

The following supporting information can be downloaded at: https://www.mdpi.com/article/10.3390/bioengineering10101206/s1, Videos S1 and S2 in the file.
